# Structural investigation of nucleophosmin interaction with the tumor suppressor Fbw7γ

**DOI:** 10.1038/oncsis.2017.78

**Published:** 2017-09-18

**Authors:** A Di Matteo, M Franceschini, A Paiardini, A Grottesi, S Chiarella, S Rocchio, C Di Natale, D Marasco, L Vitagliano, C Travaglini-Allocatelli, L Federici

**Affiliations:** 1 Istituto di Biologia e Patologia Molecolari – Consiglio Nazionale delle ricerche, Roma, Italy; 2 Dipartimento di Scienze Mediche, Orali e Biotecnologiche, Chieti, Italy; 3 CeSI-Met – Università di Chieti-Pescara ‘G d’Annunzio’, Chieti, Italy; 4 Dipartimento di Biologia e Biotecnologie ‘C Darwin’ – Sapienza Università di Roma, Roma, Italy; 5 CINECA Consorzio Interuniversitario, Sede di Roma, Roma, Italy; 6 Dipartimento di Scienze Biochimiche ‘A Rossi Fanelli’ - Sapienza Università di Roma, Roma, Italy; 7 Dipartimento di Farmacia,– Università di Napoli ‘Federico II’, Napoli, Italy; 8 Istituto di Biostrutture e Bioimmagini – Consiglio Nazionale delle Ricerche, Napoli, Italy

## Abstract

Nucleophosmin (NPM1) is a multifunctional nucleolar protein implicated in ribogenesis, centrosome duplication, cell cycle control, regulation of DNA repair and apoptotic response to stress stimuli. The majority of these functions are played through the interactions with a variety of protein partners. NPM1 is frequently overexpressed in solid tumors of different histological origin. Furthermore NPM1 is the most frequently mutated protein in acute myeloid leukemia (AML) patients. Mutations map to the C-terminal domain and lead to the aberrant and stable localization of the protein in the cytoplasm of leukemic blasts. Among NPM1 protein partners, a pivotal role is played by the tumor suppressor Fbw7γ, an E3-ubiquitin ligase that degrades oncoproteins like c-MYC, cyclin E, Notch and c-jun. In AML with NPM1 mutations, Fbw7γ is degraded following its abnormal cytosolic delocalization by mutated NPM1. This mechanism also applies to other tumor suppressors and it has been suggested that it may play a key role in leukemogenesis. Here we analyse the interaction between NPM1 and Fbw7γ, by identifying the protein surfaces implicated in recognition and key aminoacids involved. Based on the results of computational methods, we propose a structural model for the interaction, which is substantiated by experimental findings on several site-directed mutants. We also extend the analysis to two other NPM1 partners (HIV Tat and CENP-W) and conclude that NPM1 uses the same molecular surface as a platform for recognizing different protein partners. We suggest that this region of NPM1 may be targeted for cancer treatment.

## Introduction

Nucleophosmin (NPM1) is an abundant and ubiquitous protein^1^ mainly localized in nucleoli, where it contributes to their structure and organization,^2, 3^ but also shuttles between nucleolus and cytoplasm to perform its functions.^4, 5, 6^ NPM1 has a primary role in ribosome biogenesis and transport^7, 8^ but also contributes to the maintenance of genomic stability and DNA repair,^9, 10^ histones assembly,^11, 12^ centrosome duplication,^13, 14^ cell cycle regulation and response to stress stimuli.^5^ The pleiotropic behavior of NPM1 is due to its modular structure consisting of: (i) an N-terminal oligomerization domain involved in protein–protein interactions and containing two nuclear export signals (NES);^1, 4^ (ii) an intrinsically unstructured central region which contains a bipartite nuclear localization signal (NLS) and (iii) a C-terminal nucleic acid binding domain where the nucleolar localization signal (NoLS) is located.^6^ Multiple post-translational modifications such as phosphorylation, acetylation and glutathionylation regulate NPM1 localization and activities.^4, 5, 15^

NPM1 is overexpressed in several tumors, including prostate, liver, gastric, colon, pancreas, glioma and glioblastoma, astrocytoma and others.^16^ Its overexpression often correlates with mitotic index and metastatization and it was proposed as an adverse prognostic marker.^17, 18^ The *NPM1* gene is also frequently altered in hematological malignancies arising from chromosomal translocations. Here, the N-terminal domain of NPM1 is fused to protein partners such as ALK, RARα and MLF1, giving rise to oncogenic proteins and haploinsufficency for wild-type NPM1.^1^ Finally, the *NPM1* gene is the most frequently mutated in acute myeloid leukemia (AML), accounting for 30% of patients.^19^ AML mutations are localized at the C-terminal domain of the protein and cause: (i) the loss of the NoLS, (ii) a severe destabilization or the complete unfolding of the domain and (iii) the appearance of a new NES.^19^ As a result, mutated NPM1 loses affinity for nucleoli and is found stably and aberrantly in the cytoplasm.^19^

NPM1 interacts with several protein partners, modulating their stability and, importantly, it seems to have a fundamental role in their nucleolar localization. Indeed, most of NPM1 interacting proteins contain multivalent arginine-rich motifs^3^ generally found in NoLS.^20^ Furthermore, the reduction of NPM1 levels is associated with the alteration of nucleolar structure.^18^ All these aspects substantiate the hypothesis that NPM1 behaves as a hub protein in nucleoli.^2, 3^ Relevant examples of NPM1 partners include ribosomal proteins (RPL5, RPS9, RPL23), viral proteins (Rev, Tat) and many tumor suppressors, including p14ARF, p53 and Fbw7γ.^4, 16^

NPM1 is required for the nucleolar localization and stabilization of the isoform γ of Fbw7.^21^ Fbw7 belongs to the SCF (Skp1, Cullin-1, Fbox protein) class of E3-ubiquitin ligases^22^ and has a modular organization comprising: (i) the D dimerization domain, (ii) the Fbox domain that binds Skp1 of the SCF complex and (iii) the WD40 domain, which recognizes phosphorylated substrates.^22^ The *Fbw7* gene codes for three protein isoforms (namely α, β and γ) differing in their N-terminal region and displaying distinct cellular localization: Fbw7α is nucleoplasmic, Fbw7β is cytoplasmic and Fbw7γ is nucleolar.^23^ Many of Fbw7 targets are oncoproteins, including c-MYC, Notch, Cyclin E and c-Jun^22^ and therefore isoforms localization may be instrumental in their regulation through the compartmentalization of substrates recognition and degradation. For instance, nucleolar c-Myc is specifically ubiquitinylated by Fbw7γ, thus regulating its growth promoting activity.^23^

The alteration of the NPM1/Fbw7γ/c-Myc circuitry was reported in AML with *NPM1* mutations.^21^ First, it was shown that NPM1 is necessary for Fbw7γ nucleolar localization and stabilization. As a consequence, c-Myc ubiquitination and proteasome degradation is enhanced, thus lowering its levels. Conversely, c-Myc is stabilized in cells lacking NPM1 and, importantly, in AML blasts bearing the mutated form of NPM1.^21^ Indeed, mutations causing NPM1 cytoplasmic delocalization, do not compromise the interaction of NPM1 with Fbw7γ which is also delocalized in the cytoplasm and degraded.^21^ A similar delocalization/degradation mechanism was observed with the tumor suppressor p14ARF.^24, 25^ Overall, different apoptotic responses are compromised by the selective cytosolic degradation of NPM1 partners. These and other observations led to the suggestion that the NPM1 region/s implicated in protein partners recognition may be considered a target for cancer treatment.^16^

In this paper we investigate the interaction between NPM1 and Fbw7γ. We identify, in both proteins, the domains that are necessary for recognition and the aminoacids involved. We provide and validate a structural model for the interaction through protein-peptide docking and molecular dynamics simulations. We also extend this analysis to two other NPM1 interacting proteins, namely Tat^26^ and CENP-W,^27, 28^ demonstrating that the same region of NPM1 recognizes all these proteins, substantiating the proposed role of NPM1 as a ‘nucleolar hub’. We suggest that this protein region may be targeted for the treatment of AML with NPM1 mutations.

## Results

### NPM1 interacts with the predicted NoLS sequence of Fbw7γ

**Figure 1:**
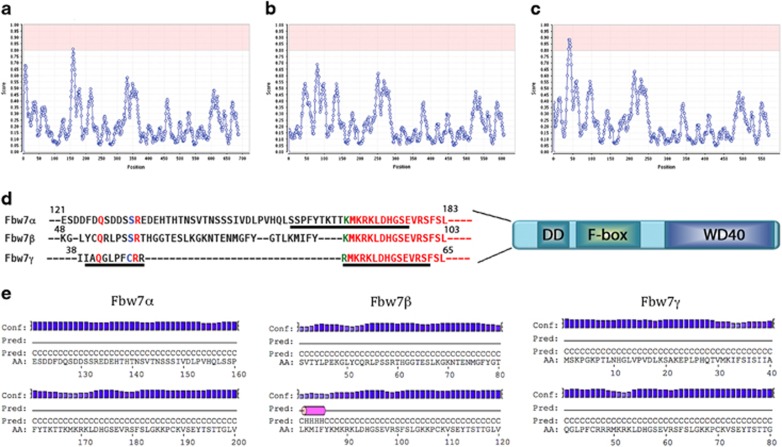
Identification of nucleolar localization signal (NoLS) in Fbw7. Fbw7 isoforms vary in their N-terminal sequence. The sequences of Fbw7α (**a**), Fbw7β (**b**) and Fbw7γ (**c**) were subjected to the NoD algorithm in order to identify putative NoLS (Scott *et al.*,^20^). The server identifies a full NoLS in Fbw7γ only (score above 0.8) while only a partial one in Fbw7α. Fbw7γ is known to be nucleolar while Fbw7α is located in the nucleoplasm. In **d** the underlined sequences correspond to the putative NoLS. PSIPRED secondary structure predictions for the three isoforms, in the regions of interest, are shown in **e**.

In an effort to understand the molecular mechanism whereby Fbw7γ localizes in nucleoli, we carried out a bioinformatic analysis of Fbw7 isoforms using the NoD algorithm (Nucleolar Localization Signal Detector; http://www.compbio.dundee.ac.uk/www-nod/index.jsp) to identify putative nucleolar localization signals (NoLS) in these proteins (Figures 1a–c). This analysis, which relies on sequence only, is based on the observation that the NoLS of many proteins consists of a short motif rich in lysines and arginines positioned in variably spaced clusters.^20^ The results showed that only the N-terminal region of Fbw7γ contains a putative NoLS (Figures 1c and d), while a partial signal is present in Fbw7α (nucleoplasmic) (Figures 1a and d) and absent in Fbw7β (cytosolic) (Figures 1b and d), in agreement with their observed cellular localization.^23^ In all three isoforms, this region of the protein is predicted to be natively unstructured (Figure 1e) and a higher amount of positive charges within few residues can be appreciated in the γ isoform with respect to the other two. Indeed the α and β isoforms contain insertion regions, separating the two clusters of positive charges found in the γ isoform, endowed with a higher conformational entropy and smaller stability, which possibly preclude their recognition as NoLS.

**Figure 2:**
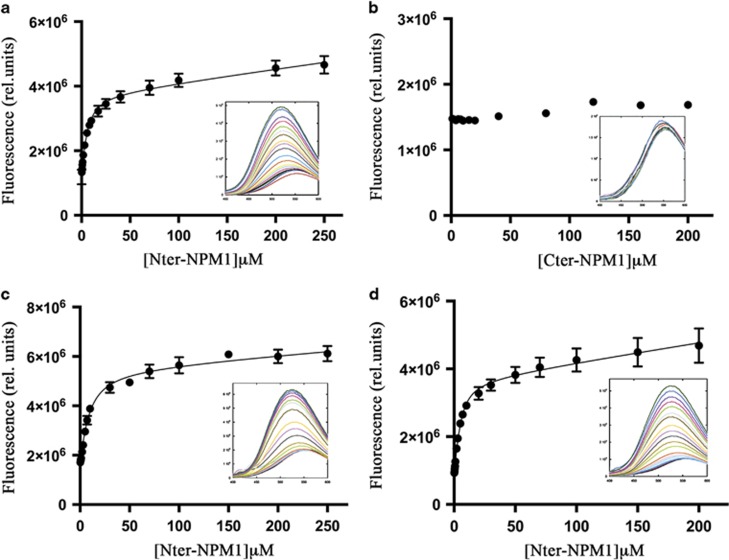
Interaction analysis of NoLS sequences. Peptides corresponding to the putative NoLS were dansylated at their N terminus and titrated with NPM1 constructs. The static fluorescence spectra are shown in insets, while the experimental maxima and their fit according to equation 1 (see Materials and Methods) are reported as a function of NPM1 concentrations in the main panels as follows: (**a**) Interaction between Fbw7γ* and Nter-NPM1. (**b**) Interaction between Fbw7γ* and Cter-NPM1. (**c**) Interaction between CENP-W* and Nter-NPM1. (**d**) Interaction between Tat* and Nter-NPM1. Peptides sequences are reported in Table 1.

Since Fbw7γ nucleolar localization depends on the presence in nucleoli of NPM1,^21^ we hypothesized that its predicted NoLS is the epitope recognized by NPM1 and we investigated the molecular determinants of this interaction. To this purpose, we used both the N-terminal and C-terminal regions of NPM1 (Nter-NPM1, residues 16-123 and Cter-NPM1, residues 225-294, respectively), and a peptide encompassing residues 43-56 of the Fbw7γ sequence (hereafter Fbw7γ*), consisting of the central region of the Fbw7γ predicted NoLS and containing six positively charged residues arranged in two clusters (Table 1). The binding process was monitored by equilibrium fluorescence spectroscopy taking advantage of a dansyl group attached to the peptide N terminus. Titrations showed an increase of fluorescence dansyl emission with a blue shift of the emission peak as a function of Nter-NPM1 concentration (see Figure 2a inset). Analysis of the data according to Equation (1) (Figure 2a) yielded an equilibrium dissociation constant *K*_D_=3.2±0.6 μm (Table 1). Data were well fitted with a 1:1 peptide/Nter-NPM1 monomer stoichiometry, even though Nter-NPM1 is pentameric at all concentrations tested in our experiments.

When the C-terminal domain of NPM1 (Cter-NPM1) was used, no variation in the emission peak was observed, indicating that it does not bind Fbw7γ* (Figure 2b). We also performed the same experiments using dansylated peptides (named unrelated long and unrelated short) whose sequences are not present in the Fbw7γ isoform and which are not predicted to be NoLS (Table 1). No interaction was observed when these peptides were titrated with Nter-NPM1.

Since it has been reported that NPM1 interacts also with CENP-W and Tat proteins,^26, 27^ we used the NoD algorithm to identify in these two proteins their NoLS. Then, dansylated peptides corresponding to the suggested NoLS regions of the proteins (peptides CENP-W* and Tat*, respectively) (Table 1) were tested for their interaction with Nter-NPM1 (Figures 2c and d) and Cter-NPM1 (Supplementary Figure 1). For both peptides, equilibrium titrations experiments showed an increase of fluorescence emission as a function of protein concentration only when the Nter-NPM1 domain was used (Figures 2c and d), demonstrating that the interaction specifically involves the N-terminal domain and that, also in these cases, the predicted NoLS is the binding epitope recognized by NPM1. The calculated dissociation constants parallel the one obtained for Fbw7γ, being *K*_D_ =6.2±0.9 μm for the Nter-NPM1-CENP-W* interaction and *K*_D_ =2.4±0.5 μm for the Nter-NPM1-Tat* interaction (Table 1).

### Identification of Nter-NPM1 residues involved in Fbw7γ recognition

**Figure 3:**
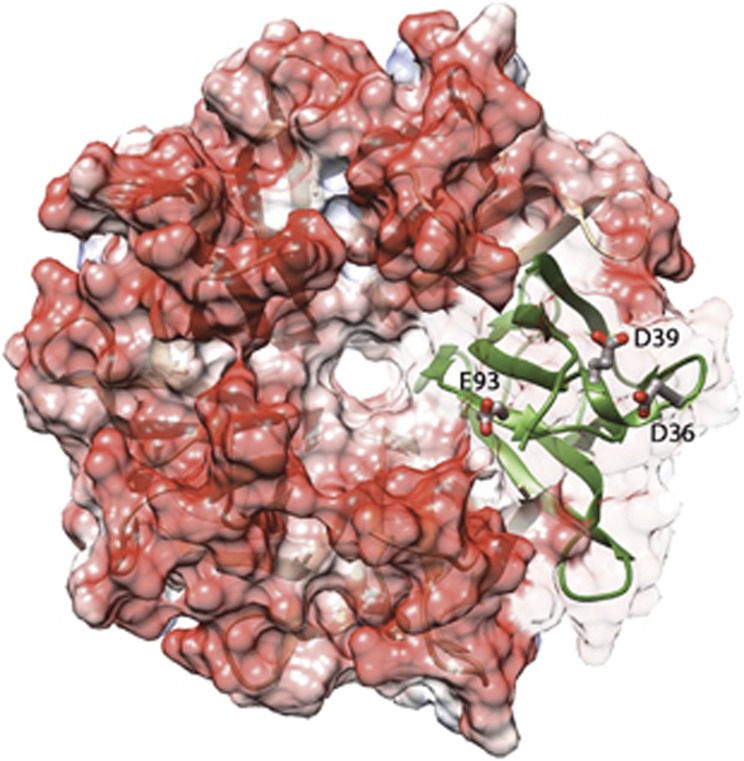
Electrostatic potential surface analysis of Nter-NPM1. The crystal structure of human Nter-NPM1 displays a pentameric organization and was subjected to calculation of the electrostatic potential surface through the APBS algorithm. Negative and positive charges are shown in red and blue, respectively. One of the monomers is shown in ribbon to better highlight the position of three important acidic residues (D36, D39 and E93), which are shown in sticks.

Nter-NPM1 monomer consists of eight antiparallel β-strands forming a β-barrel with jelly-roll topology. Five monomers tightly associate to form a crown shaped pentamer. Since the identified Nter-NPM1-interacting epitopes are all enriched in positive charges, we inspected the electrostatic potential surface of Nter-NPM1 in search for negatively charged patches. Indeed, as shown in Figure 3, a large negatively charged surface is found, extending from the pentamer external surface to its central cavity. Among the residues that contribute to this negatively charged surface, we focused our attention on three residues, namely D36, E39 and E93, because they were previously shown to play a role in the interaction of NPM1 with the tumor suppressor p14ARF.^29^ To establish their involvement in binding Fbw7γ*, these residues were all mutated to alanine, as single or double mutants; a triple mutant was also prepared. The interaction was measured using the same protocol as for the wild-type protein (Supplementary Figure 2) and the resulting *K*_D_ are reported in Table 2. Mutation to alanine of D36 and E39 residues led to an increase of *K*_D_ between two and three fold. Mutation of E93 had instead a smaller effect. Consistently, when the double mutants D36A-E93A and E39A-E93A where tested, observed *K*_D_ were comparable to those obtained with the D36A and E39A single mutants, respectively. When the D36A-E39A double mutant was tested no dramatic additive effect of the two mutations in destabilizing the interaction was obtained with *K*_D_ =13.5±2.0 μm. Finally, the triple mutant D36A-E39A-E93A resulted to bind the peptide with a *K*_D_ =22±3 μm, around seven fold higher than wild type. These data suggest that though residues D36, E39 and to a minor extent E93, contribute to the binding energy, the overall binding process is not entirely dependent on their interactions. Additional residues are likely involved.

In order to obtain a better description of the interaction and identify additional residues involved, we performed a molecular docking analysis of the Nter-NPM1- Fbw7γ* complex. Given the complexity of docking a long and flexible peptide, the procedure adopted here was based on a ‘divide and conquer’ approach, starting from a combinatorial merging of energy-favorable tripeptides, which were then used as templates for biased-guided docking (see Materials and Methods for a detailed description). The top ten scoring docking poses cluster in the same binding site, with a mean RMSD between poses of 4.3 Å (Supplementary Figure 3). This is principally the result of the variable conformations adopted by the C-terminal half of the peptide, while the N-terminal half appears more fixed. Only a single peptide was docked onto the Nter-NPM1 pentamer, nevertheless equivalent docking surfaces are available for the additional peptides. It is possible that different peptide conformations, among those selected by the docking procedure, would be adopted when all Nter-NPM1 monomers are engaged by peptides.

**Figure 4:**
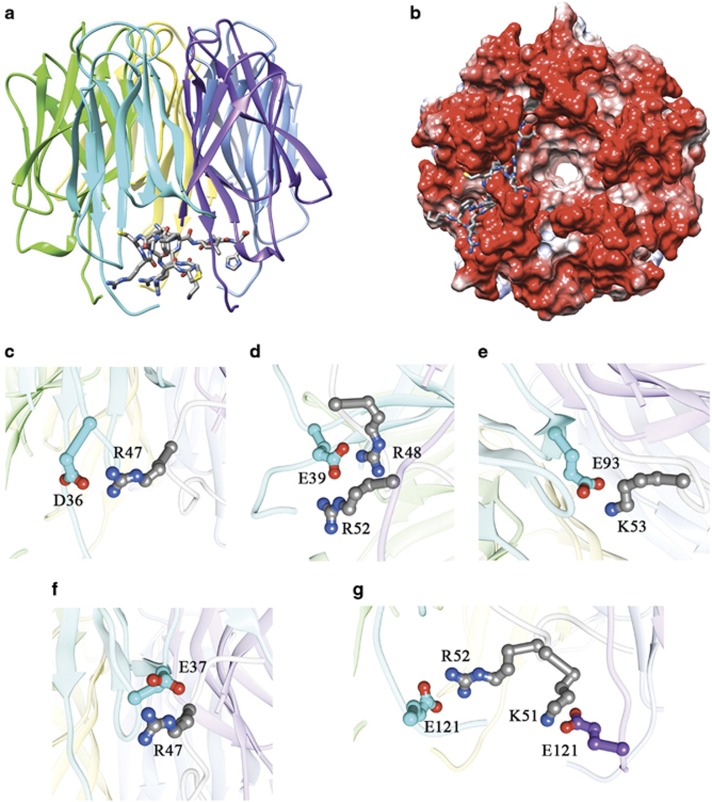
Molecular docking analysis of the Fbw7γ*-Nter-NPM1 interaction. The interaction between Fbw7γ* and Nter-NPM1 investigated through molecular docking analysis is shown. (**a**) Nter-NPM1 pentamer is represented in cartoon while the Fbw7γ* peptide is shown in sticks. (**b**) The Nter-NPM1 electrostatic surface is shown in a different orientation from **a**. The peptide, shown in sticks, adopts an extended conformation with its C-terminal end protruding into the central pentamer cavity. (**c**) A detail of the interaction played by Nter-NPM1 residue D36 with Fbw7γ* R47. (**d**) Nter-NPM1 residue is predicted to interact with both Fbw7γ* R48 and R52 residues. (**e**) Interaction between Nter-NPM1 E93 and Fbw7γ* K53. (**f**) Nter-NPM1 residue E37 is also predicted to interact with Fbw7γ* R47. (**g**) E121 residues from two different Nter-NPM1 monomers (the second one is shown in magenta) are predicted to interact with residue K51 and R52 residues.

Figures 4a and b show the best scoring docking pose and, according to this model, Fbw7γ* adopts an extended conformation and lies with its N-terminal end along the external surface of the protein while its C-terminal end protrudes into the central cavity of the pentamer. The majority of interactions are established with residues belonging to a single Nter-NPM1 monomer, with few contributions from the adjacent one (see below). Interestingly, although no information regarding interacting residues was imparted to the docking algorithm, all three residues that we have examined before (D36, D39 and E93) were found to interact with positively charged residues of the Fbw7γ* peptide. In particular, in the docking model, D36 forms a salt bridge with Fbw7γ* R47 (Figure 4c), E39 is salt-bridged to both R48 and R52 (Figure 4d), while E93 interacts with K53 (Figure 4e).

Inspection of the model showed two additional negatively charged residues that interact with Fbw7γ*. The first one is E37 that interacts with R47 (Figure 4f); the second one is E121: in this case the same residue from two different Nter-NPM1 monomers binds two different residues of the peptide, i.e., K51 and R52 (Figure 4g). Therefore, we mutated these two additional residues and measured their contribution to the binding energy (Supplementary Figure 2). When E37A and E121A single mutants were tested, we obtained dissociation constants approximately four-fold higher than wild-type, similarly to what already seen for the D36 and E39 mutants (Table 2). Furthermore, starting from the D36A-E39A-E93A triple mutant, we also prepared two quadruple mutants and a quintuple one. When the D36A-E37A-E39A-E93A mutant was tested, we obtained a *K*_D_ =151.5±20.5 μm, ≈50-fold higher than wild type. Likewise, the D36A-E39A-E93A-E121A mutant showed a *K*_D_ =224.3±35.9 μm, ≈70-fold higher than wild type. Finally, the quintuple mutant displayed negligible affinity for the peptide, with *K*_D_ =653.7±46.9 μm (Table 2).

Overall, these data suggest that the Nter-NPM1-Fbw7γ* binding energy is dictated by multiple electrostatic contributions throughout the binding cleft. They also show a non-linear variation of the *K*_D_ upon loss of negative charges in Nter-NPM1. In fact the dissociation constant is relatively stable when only three residues are mutated, while it markedly increases upon addition of a fourth mutation. To check the effect of salt on binding we also performed titrations with wild-type protein and triple, quadruple and quintuple mutants increasing the ionic strength to 150 mm. We obtained a general decrease in affinity, as expected, but the trend observed with the previous experiments was confirmed (Supplementary Table 1).

### The same surface of Nter-NPM1 recognizes peptides from different protein partners

Since the peptides from CENP-W and Tat recognized by Nter-NPM1 share with Fbw7γ* a high positive charge (Table 1), we investigated whether the same Nter-NPM1 surface is implicated in their binding. To this end, we tested the triple, quadruple and quintuple Nter-NPM1 mutants interaction with the CENP-W* and Tat* peptides (Supplementary Figure 4). In both cases, and similarly to what already seen for Fbw7γ*, a clear trend of increasing dissociation constants is observed as a function of decreasing negative charges from the triple to the quintuple mutant (Table 3). These results indicate that the same region of Nter-NPM1 is responsible for the interaction with different nucleolar proteins that are all recognized through their predicted NoLS.

### Molecular dynamics simulations

**Figure 5:**
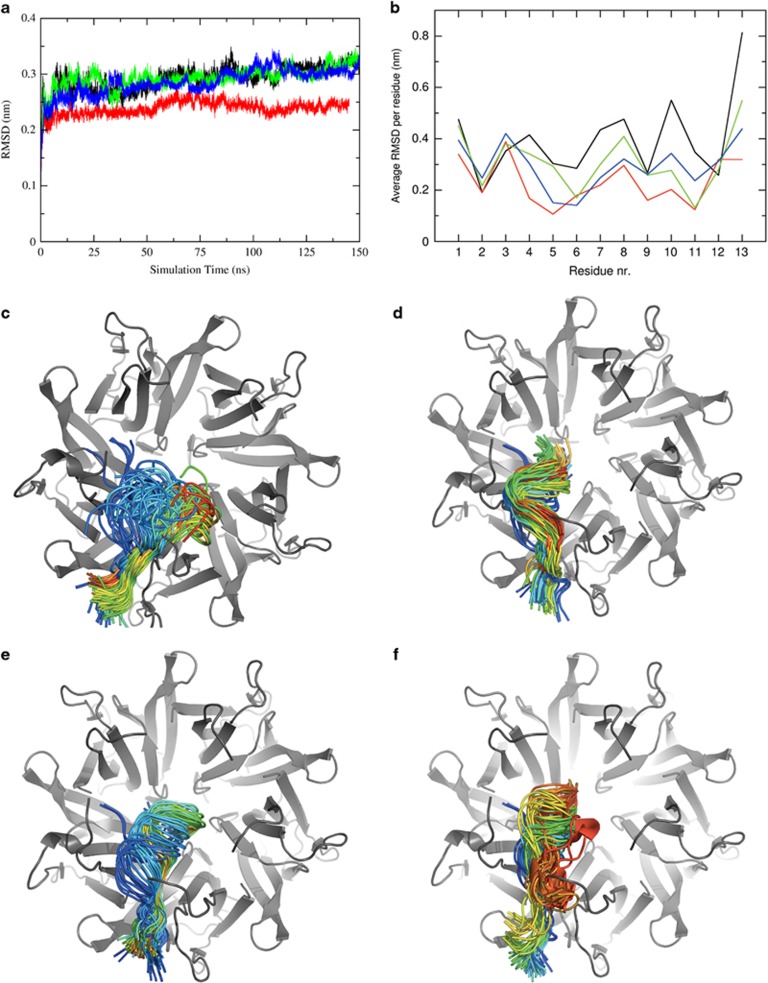
Molecular dynamics simulations of the interaction between Fbw7γ* and Nter-NPM1 constructs (wild-type and mutants). (**a**) Root mean square deviation (RMSD) of Nter-NPM1 and Fbw7γ* Cα atoms as a function of simulation time for WT (black line), triple (red), quadruple (green) and quintuple (blue), respectively. (**b**) Average RMSD (root mean square deviation) of Fbw7γ* as calculated for peptide Cα residues for all simulated systems. Wild-type Nter-NPM1 is shown in black, the triple D36A-E39A-E93A mutant is shown in red, the quadruple D36A-E39A-E93A-E121 mutant is shown in green, the quintuple D36A-D37A-E39A-E93A-E121 is shown in blue. (**c**) The position of the peptide at selected snapshots along the simulation time is shown. Nter-NPM1 wild-type is shown in gray cartoon, Fbw7γ* is shown as a ribbon colored from blue (simulation start time) to red (simulation end). (**d**) Same as in **c** for the interaction between the peptide and the triple D36A-E39A-E93A mutant. (**e**) Same as in **c** for the interaction between the peptide and the quadruple D36A-E39A-E93A-E121 mutant. (**f**) Same as in **c** for the interaction between the peptide and the quintuple D36A-D37A-E39A-E93A-E121 mutant.

To gain further insights into the structural requirements for binding, we performed extended molecular dynamics (MD) simulations on the model structure for the complex between Fbw7γ* and Nter-NPM1. Given their increasing effect on the dissociation constant of the complex, we simulated also the complexes formed by Fbw7γ* with the D36A-E39A-E93A triple mutant, the D36A-E39A-E93A-E121A quadruple mutant and the D36A-E37A-E39A-E93A-E121A quintuple mutant. Total simulation time for all systems was 150 ns. We firstly determined the convergence and stability of the MD trajectories, in order to ascertain the validity of conformational sampling in all simulated systems. To this end, the root mean square deviations (RMSD) of Cα coordinates of wild type and mutants Nter-NPM1 were calculated as a function of simulation time (Figure 5a). This analysis confirmed that the trajectories reached a plateau of the RMSD, a regime compatible with the conformational drift of a folded structure and that the simulation time was sufficient to equilibrate the protein dynamics.

This enabled us to investigate the nature of the Nter-NPM1-Fbw7γ* interaction by analyzing the relative conformational drift of Fbw7γ* with respect to the Nter-NPM1 and mutants structures. Figure 5b shows the averaged RMSD per peptide residue, as calculated for Cα atoms, for all simulated systems. This analysis suggests that the Fbw7γ* peptide is stabilized in the binding surface of wild-type Nter-NPM1 through interactions involving mainly its N-terminal residues 1–6, which keep a position similar to the starting structure throughout the whole simulation. This is also represented in Figure 5c, showing snapshots of the Nter-NPM1-Fbw7γ* simulation, with the conformation adopted by the peptide at different times from the beginning (blue) to the end (red) of the simulation. Differently from the N-terminal end, the C-terminal region of the peptide, which protrudes inside the central cavity of the Nter-NPM1 pentamer, populates several conformations along the simulation time (Figure 5c). Overall, the interaction of wild-type Nter-NPM1 with Fbw7γ* has the highest RMSD values as compared with the mutants (Figure 5b). It appears that no single ion pair is absolutely required for the interaction because adjacent negative residues can replace the loss of a contact. We speculate that such binding mode allows the stabilization of the peptide into the cleft without a significant entropy loss, since many energy minima can be efficiently explored by the peptide (Figure 5c). This also explains why single and double NPM1 mutants show only a weak decrease of the binding affinity for Fbw7γ*.

The analysis of the RMSD over peptide length for the triple, quadruple and quintuple mutants suggest that in all mutants the positions explored by the peptide are less variable as compared with wild type, as shown in Figure 5b which represents a measure of the average displacement of each peptide Cα atom with respect to its starting position. Indeed, snapshots of the simulations at different times indicate that the peptide docks into the binding surface of the mutants maintaining an overall less variable conformation along the simulation time (Figures 5d–f). This observation can be rationalized by taking into account the hydrophobic interactions played by the newly introduced alanine residues in the mutants, which are favored and replace many of the electrostatic interactions previously observed for the wild-type protein.

Overall we hypothesize that while Fbw7γ* is still able to bind all NPM1 mutants, the sequential loss of negative charges may be associated with a significant entropy loss upon binding and a progressively decreased affinity.

## Discussion

In this work we investigated the structural basis of NPM1 protein–protein associations. We started from the hypothesis that NPM1 is a ‘nucleolar hub’ protein because it recognizes the NoLS of different protein partners. In most proteins, the NoLS consists of a linear stretch of aminoacids, within a natively disordered region, that is rich in clustered arginines and lysines. Even though a clear sequence motif cannot be envisaged, such accumulation of positive charges within few residues is uncommon in proteins and may be searched for by specific algorithms. We employed one such algorithm to spot putative NoLS in representative proteins that are known to interact with NPM1 and to be nucleolar. Then, we showed that all these epitopes from different proteins were effectively recognized by Nter-NPM1.

A deeper analysis of the interaction highlighted additional concepts. First, we showed that at least five negatively charged residues of Nter-NPM1 contact the peptides. Then, by mutating them alone and in combinations, we could argue against the existence of specific hot-spots. Rather, we observed a substantial stability of the complex when negative charges where replaced alone or in couples, and appreciated gradually increasing dissociation constants when three to five residues where mutated at once. Molecular dynamics simulations provided a plausible mechanism to interpret these observations and suggested that the absence of hot-spots is the consequence of the fact that the loss of one interaction may be compensated by the emergence of a new one. This is possible because the peptide does not stably populate a single conformation but moves rather freely within an extended binding surface provided by Nter-NPM1. Therefore the loss of a single or a couple of ion-pairs that would destabilize one conformation may be compensated by the adoption of an alternative one by the peptide.

Such a model may also explain the amazing versatility of Nter-NPM1 in binding epitopes from a plethora of other proteins. The peptides from Fbw7γ, TAT and CENP-W that we identified here, as well as several other peptides studied by others,^3, 29, 30^ are all positively charged but differ in residue composition, in the number of charged residues and in their position along the sequence. How can a single protein recognize them all with similar affinities? The model we propose implies that all peptides will find, within the large negatively charged surface provided by the NPM1 pentamer, a multitude of binding poses and will populate those that are more convenient to their particular distribution of positive charges. We speculate that such mechanism is at the heart of NPM1 behavior as a nucleolar hub protein.

But how NPM1 is itself enriched in nucleoli? Previous research from our and other laboratories has clarified this issue. The NPM1 nucleolar localization signal is unique and consists of W288 and W290 residues near the C terminus of the protein. These tryptophans take part to the hydrophobic core of the C-terminal three-helix bundle domain and their substitution with other residues leads to the unfolding of the C-terminal domain.^31, 32, 33, 34^ An unfolded C-terminal domain is in turn unable to interact with nucleic acids, most prominently G-quadruplex regions at ribosomal DNA, resulting in detachment from nucleoli.^35, 36, 37^ Therefore, the C-terminal domain of NPM1 keeps it at nucleoli while the N-terminal domain sequesters its binding partners in the same organelle. When NPM1 moves from nucleoli, because of post-translational modifications or mutations, the NPM1 protein partners will be equally displaced, because of their interaction with the N-terminal domain.

This is ultimately what happens in AML with *NPM1* gene mutations. Mutations cause the unfolding of the C-terminal domain and consequent loss of affinity for nucleoli. Furthermore, since a new NES appears in the mutated protein, this is aberrantly translocated in the cytosol, carrying with itself protein partners like Fbw7γ and p14ARF, which will be there degraded.^21, 24^ Moreover, the presence in the cytosol of mutated NPM1 with a functional N-terminal domain, will result in the establishment of additional protein–protein interactions. For instance, cytosolic NPM1 binds and inhibits caspases 6 and 8^38^ and the PTEN deubiquitinating enzyme HAUSP, resulting in PTEN cytoplasmic polyubiquitinilation and degradation.^39^ Thus a third important tumor suppressor is also deregulated by the presence of NPM1 in the cytosol.

AML with *NPM1* mutation is currently treated with the administration of several cycles of an anthracycline (daunorubicin, doxorubicin or others) plus cytarabine.^40^ Patients carrying *NPM1* mutation without the concomitant *FLT3-ITD* alteration have good prognosis while, for the latter, chemotherapy is less effective. However, relapse is frequent and the toxicity of anthracyclines prevents many patients from its prolonged use. Importantly, NPM1 mutations are always retained at relapse and this led to the generally accepted concept that NPM1 should be specifically targeted in this kind of leukemia.^41^

Based on the experimental observations we outlined above, we have recently suggested that an effective strategy to specifically target AML with *NPM1* mutations would be that of interfering with NPM1 protein–protein interactions.^16^ Here, we have characterized the extended surface of Nter-NPM1 involved in protein binding and thus provided a structural framework to search for small molecules and/or peptidomimetics targeting this surface. Future work will be directed at testing these concepts in cellular models of AML with *NPM1* mutations.

## Materials and methods

### NoLS identification

To identify NoLSs in the proteins of interests to this work we employed the method described by Scott *et al.*^20^ and implemented in the NoD web server (http://www.compbio.dundee.ac.uk/www-nod/). Briefly, the NoD algorithm uses an artificial neural network trained on a large set of NoLs experimentally evaluated, to analyze a sequence in search of local enrichments of positively charged residues. The sequence of a protein is scanned in windows of 13 residues with slippage of one amino acid for each consecutive window and at each window is assigned a score, which depends on the number of positive charges. When the score is greater than 0.8, the relative sequence is identified as a predicted NoLs.

### Protein constructs

The Nter-NPM1 (residues 16-123) coding sequence was obtained through gene synthesis (GeneArt, Regensburg, Germany) and cloned into the expression vector pET28+(a) (Novagen, San Diego, CA, USA) using NdeI and BamHI restriction enzymes.

Nter-NPM1 mutants were obtained by site-directed mutagenesis using the Quickchange II Lightning Site-Directed Mutagenesis kit (Stratagene, La Jolla, CA, USA), following manufacturer’s instructions. Oligonucleotides used for PCR were obtained from Primm Biotech (Milan, Italy). Forward oligonucleotides used are reported in Supplementary Table 2.

### Protein expression and purification

Escherichia coli cells, BL21(DE3) (Biolabs, Ipswich, MA, USA), transformed with the expression vectors were grown to A_600_ ~0.5 in LB medium supplemented with kanamycin at 37 °C. Expression was induced by addition of 1 mm isopropyl-1-thio-β-d-galactopyranoside (IPTG) and cells were further grown at 20 °C for 16h. Cells were collected, resuspended in lysis buffer (Buffer A: 20 mm Hepes, pH 7.0, 20 mm imidazole), plus 5 mm MgCl_2_, 2 μg/ml DNAse (Roche, Basel, Switzerland), Protease Inhibitor Cocktail Tablet (Roche) and sonicated. Nucleic acids were digested for 30’ at 37 °C with DNAse I. Proteins were purified by affinity chromatography (HisTrap FF, GE Healthcare, USA) using a linear gradient of buffer A plus imidazole (from 20 mm to 1m). Further purification involved anion exchange chromatography (Q-Sepharose Fast Flow, GE Healthcare, USA) eluted with NaCl gradient. Fractions containing the protein, as showed by SDS–PAGE, were collected and concentrated using Amicon Ultra-15 Centricons with a 3K cut-off (Merck Millipore, Darmstadt, Germany). The protein solutions were buffer exchanged with Hepes 20 mm, pH 7.0 and stored at −20 °C. The Cter-NPM1 protein construct (residues 225-294) was expressed and purified as previously described.^36^

### Equilibrium binding experiments

All experiments were performed at 25 °C in sodium phosphate buffer 20 mm pH 7.2, or in sodium phosphate buffer 20 mm plus 100 mm NaCl pH 7.2 (which sets ionic strength to 150 mm), using a FluoroMax-4 spectrofluorometer (Jobin Yvon, Edison, NJ, USA), equipped with a water bath apparatus. Fbw7γ and CENP-W peptides, functionalized with a dansyl (5-dimetilamminonaftalen-1-sulphonyl) group at their N terminus, were purchased from JPT (Germany). Tat peptide was synthesized employing the solid phase method following standard Fmoc strategies and labeled with dansyl fluorophore at its N terminus. Crude product was purified by RP-HPLC applying a linear gradient of 0.1% TFA CH_3_CN in 0.1% TFA water from 5% to 65% over 12 min using a semi-preparative 2.2 × 5 cm C18 column at a flow rate of 20 ml/min. Purity and identity were confirmed by LC–MS analysis.

Titration experiments were conducted with an excitation wavelength of 330 nm while the fluorescence emission spectra were collected in the range between 350 and 650 nm. Titrations were performed at constant peptide concentration (5 μm) and varying protein concentrations (from 0 to 200/400 μm). Titrations were performed in triplicate and data, analyzed with the Graphpad Prism software (https://www.graphpad.com/scientific-software/prism/), were reported as dissociation constant±s.d.

Equilibrium binding curves were fitted to the standard quadratic equation:


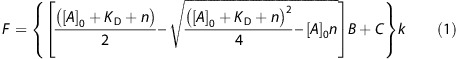


where *F* is the observed fluorescence signal, *n* and [*A*]_0_ are the total concentration of non-varied and varied species, respectively, and *K*_D_ is the equilibrium dissociation constant. *B* and *C* are constants taking into account the total fluorescence change and fluorescence at [*A*]_0_=0, respectively, and *k* is a term describing the slope of the curve at high protein concentration^42^. Whenever possible, under pseudo first-order conditions, the equation 1 was simplified to:


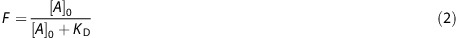


### Molecular docking

In order to predict the binding mode of the peptide N_ter_-PFCRRRMKRKLDH-C_ter_ to NPM1, tripeptides covering the whole sequence were exhaustively generated by an ad-hoc Python script.^43^ The obtained peptides were then energy minimized by using the Molecular Operating Environment 2009.10 (http://www.chemcomp.com/MOE-Molecular_Operating_Environment.htm). Steepest descents steps of energy minimization were performed until the root mean square (RMS) gradient fell below the 0.005 Å default threshold. The Amber99 force field, a distance-dependent dielectric constant and a cut-off distance of 40 Å were used during each simulation.

Molecular docking of the tripeptides was carried out by means of Molegro Virtual Docker (MVD) software (®CLCbio).^44^ Flexible torsions were automatically detected by MVD, and manually checked for consistency. The structure of NPM1 (PDB: 2P1B) was prepared by automatically assigning bond orders and hybridization, and adding explicit hydrogens, charges and Tripos atom types. Missing heavy atoms were fixed by modeling them, using Modeler v.9.8^45^ and PyMod.^46^ A search space of 20Å radius, centered on the central cavity of the pentamer (∼4385 Å^3^) was used for docking. Cavity detection was carried out by MVD. For each tripeptide, ten runs were defined. Similar poses (RMSD <1.2 Å) for each tripeptide were clustered, and the best scoring one was taken as representative. Other docking parameters were fixed at their default values. Thereafter, hexamer peptide sequences and their structures were generated and docked in a second round, by considering the poses of the tripeptides identified from the first round of docking runs. To this end, the most energetically favorable poses of the tripeptides were taken as template groups for template-based dockings. Finally, the 100 top scoring hexamer peptides poses were taken as template groups for template-based docking of the whole peptide fragment, using the same above-described approach. The obtained top scoring complex was subjected to a final energy minimization, using conjugated gradients until the maximum derivative was less than 0.0004 kJ mol^−1^Å^−1^.

### Molecular dynamics simulations

MD simulations of complexes were performed starting from the final refined model obtained in docking calculations. Mutations were introduced with the Pymol software (www.pymol.org).

#### Simulation setup

Calculations were performed using GROMACS 5.0.x (www.gromacs.org) suite with the Amber99 force field. Initial structures were immersed in a triclinic simulation box, solvated with SPC water molecules.^47^ Ionic strength was adjusted to set the total charge of simulation box to 0. All simulations were performed in the NVT ensemble at constant volume and constant temperature (300 K), periodic boundary conditions were applied. Initial velocities were taken from the Maxwell-Boltzmann distribution at 300 K. Long-range electrostatic interactions were calculated using the particle mesh Ewald method^48^ with a 1.2 nm cut-off for the real space calculation. A 1.2 nm cut-off was used to estimate Van der Waals interactions. Pair list was updated every 10 steps. The LINCS algorithm^49^ was used to constrain bond lengths; the time step for integration was 2 fs.

#### Simulation protocol

Intial structures for all simulations were subjected to a steepest descent minimizzation cycle to reduce steric hindrance. Then a restrained MD step-wise procedure was applied to gradually release the restraints and allow the system to equilibrate at the simulated temperature of 300 K: applied restrained were 1000, 500 and 250 kJ/mol. Total simulation time was typically 150 ns. Coordinates were saved every 5 ps.

## Publisher's note:

Springer Nature remains neutral with regard to jurisdictional claims in published maps and institutional affiliations.

## Figures and Tables

**Table 1:** Dissociation constants for the complexes between Nter-NPM1 and peptides

*Peptide*	*Sequence*	*Nter-NPM1* K_*D*_*(μm)*
Fbw7γ*	^43^LPFCRRRMKRKLDH^56^	3.2±0.6
CENP-W*	^14^KRKAPRGFLKRVFKRKK^30^	6.2±0.9
Tat*	^47^AGRKKRRQRRRPPQ^60^	2.4±0.5
Unrelated long	DDEAQTLAKFVLSQK	Ni
Unreleted short	VLSQK	Ni

Abbreviation: Ni, no interaction.

**Table 2:** Dissociation constants for the complexes between the Fbw7γ* peptide and Nter-NPM1 mutants

*Protein*	K_*D*_ *(μm)*
Nter-NPM1	3.2±0.6
D36A	10.8±2.6
E37A	12.5±1.4
E39A	6.2±1.5
E93A	5.0±1.6
E121A	13.3±3.8
D36A-E39A	13.5±2.0
D36A-E93A	8.4±1.5
E39A-E93A	7.6±1.9
D36A-E39A-E93A	22.0±3.0
D36A-E37A-E39A-E93A	151.5±20.5
D36A-E39A-E93A-E121A	224.3±35.9
D36A-E37A-E39A-E93A-E121A	653.7±46.9

**Table 3:** Dissociation constants for the complexes between Nter-NPM1 selected mutants and the CENP-W* and Tat* peptides

	CENP-W* K_*D*_ *(μm)*	Tat* K_*D*_ *(μm)*
Nter-NPM1	6.2±0.9	2.4±0.5
D36A-E39A-E93A	18.4±2.6	11.1±2.1
D36A-E37A-E39A-E93A	71.4±9.1	57.4±7.0
D36A-E39A-E93A-E121A	82.8±5.6	63.8±5.0
D36A-E37A-E39A-E93A-E121A	734.0±158.0	642.6±70.8
