# Supplementary material for: Structural investigation of nucleophosmin interaction with the tumor suppressor Fbw7γ
Source: Oncogenesis. 2017 Sep 18;6(9):e379–. doi: 10.1038/oncsis.2017.78 (PMC5623904; doi:10.1038/oncsis.2017.78)
Supplement: Supplementary Table 1 [file oncsis201778x1.docx]

| **Protein** | **K_D_ (µM)**  **100mM NaCl** |
| --- | --- |
| Nter–NPM1 | 20.6± 2.6 |
| D36A-E39A-E93A | 66.8± 12.5 |
| D36A-E37A-E39A-E93A | 157.5± 12.3 |
| D36A-E37A-E39A-E93A-E121A | No Interaction |

**Table S1.** Interaction between NPM1-Nter and Fbw7γ* at 150 mM ionic strength
