# Supplementary material for: Structural investigation of nucleophosmin interaction with the tumor suppressor Fbw7γ
Source: Oncogenesis. 2017 Sep 18;6(9):e379–. doi: 10.1038/oncsis.2017.78 (PMC5623904; doi:10.1038/oncsis.2017.78)
Supplement: Supplementary Table 2 [file oncsis201778x2.docx]

| Mutant | Vector | Forward sequence (5’ 🡪 3’) |
| --- | --- | --- |
| D36A | pET28(a)^+^NPM1-Nter | CCATTTCAAAGTGGATAAC**GCG**GAAAACGAACATCAGCTG |
| E37A | pET28(a)^+^NPM1-Nter | AAAGTGGATAACGAT**GCG**AACGAACATCAGCTG |
| E39A | pET28(a)^+^NPM1-Nter | GGATAACGATGAAAAC**GCG**CATCAGCTGTCTCTGC |
| E93A | pET28(a)^+^NPM1-Nter | CCTGGGCGGCTTT**GCG**ATTACTCCGCCGG |
| E121A | pET28(a)^+^NPM1-Nter | CTGGTGGCGGTGGAA**GCG**GATGCGTAATAAGG |
| D36A-E39A | pET28(a)^+^NPM1-Nter-D36A | GATAACGCGGAAAAC**GCG**CATCAGCTGTCTCTG |
| D36A-E93A | pET28(a)^+^NPM1-Nter-D36A | CCTGGGCGGCTTT**GCG**ATTACTCCGCCGG |
| E39A-E93A | pET28(a)^+^NPM1-Nter-E39A | CCTGGGCGGCTTT**GCG**ATTACTCCGCCGG |
| D36A-E39A-E93a | pET28(a)^+^NPM1-Nter-D36A-E39A | CCTGGGCGGCTTT**GCG**ATTACTCCGCCGG |
| D36A-E37A-E39A-E93A | pET28(a)^+^NPM1-Nter- D36A-E39A-E93A | AAAGTGGATAAC**GCGGAT**AAC**GCG**CATCAGCTG’ |
| D36A-E39A-E93A-E121A | pET28(a)^+^NPM1-Nter- D36A-E39A-E93A | CTGGTGGCGGTGGAA**GCG**GATGCGTAATAAGG |
| D36A-E37A-E39A-E93A-E121A | pET28(a)^+^NPM1-Nter- D36A-E37A-E39A-E93A | CTGGTGGCGGTGGAA**GCG**GATGCGTAATAAGG |

**Supplementary Table 2.** Oligonucleotides used to prepare Nter-NPM1 site-directed mutants
