# Supplementary figures and images for: Structural investigation of nucleophosmin interaction with the tumor suppressor Fbw7γ
Source: Oncogenesis. 2017 Sep 18;6(9):e379–. doi: 10.1038/oncsis.2017.78 (PMC5623904; doi:10.1038/oncsis.2017.78)

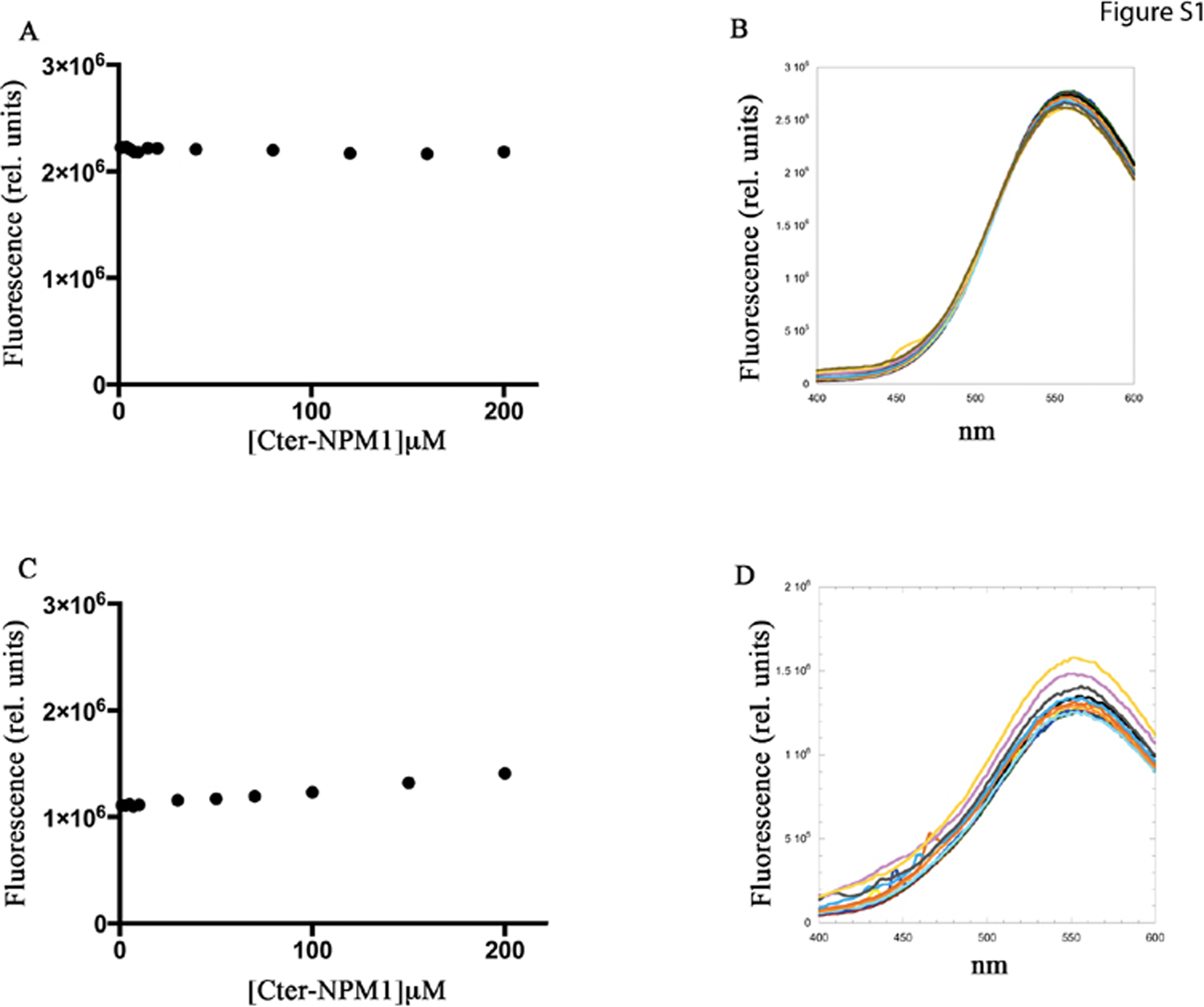

Supplement: Supplementary Figure 1 [file oncsis201778x3.tif]

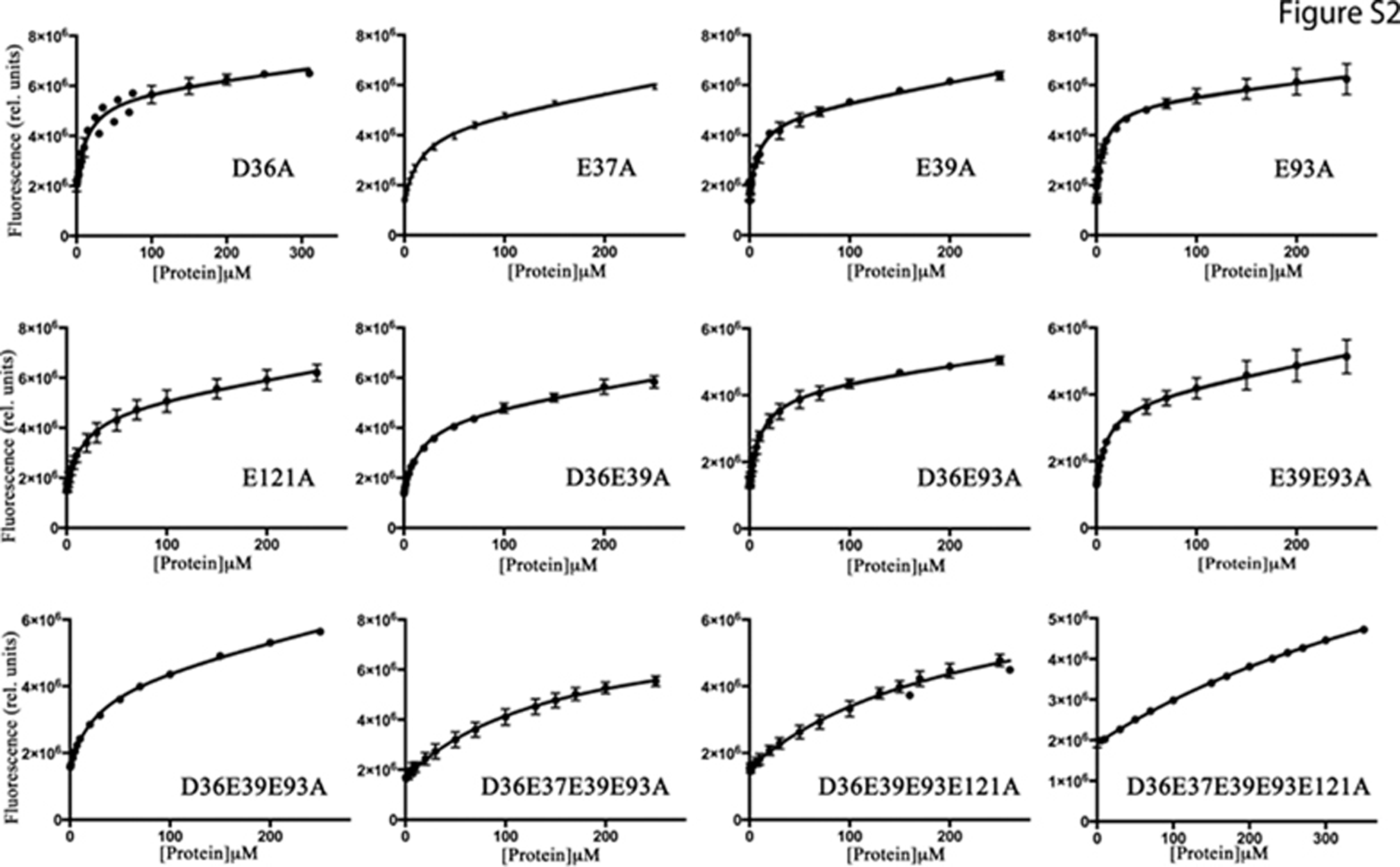

Supplement: Supplementary Figure 2 [file oncsis201778x4.tif]

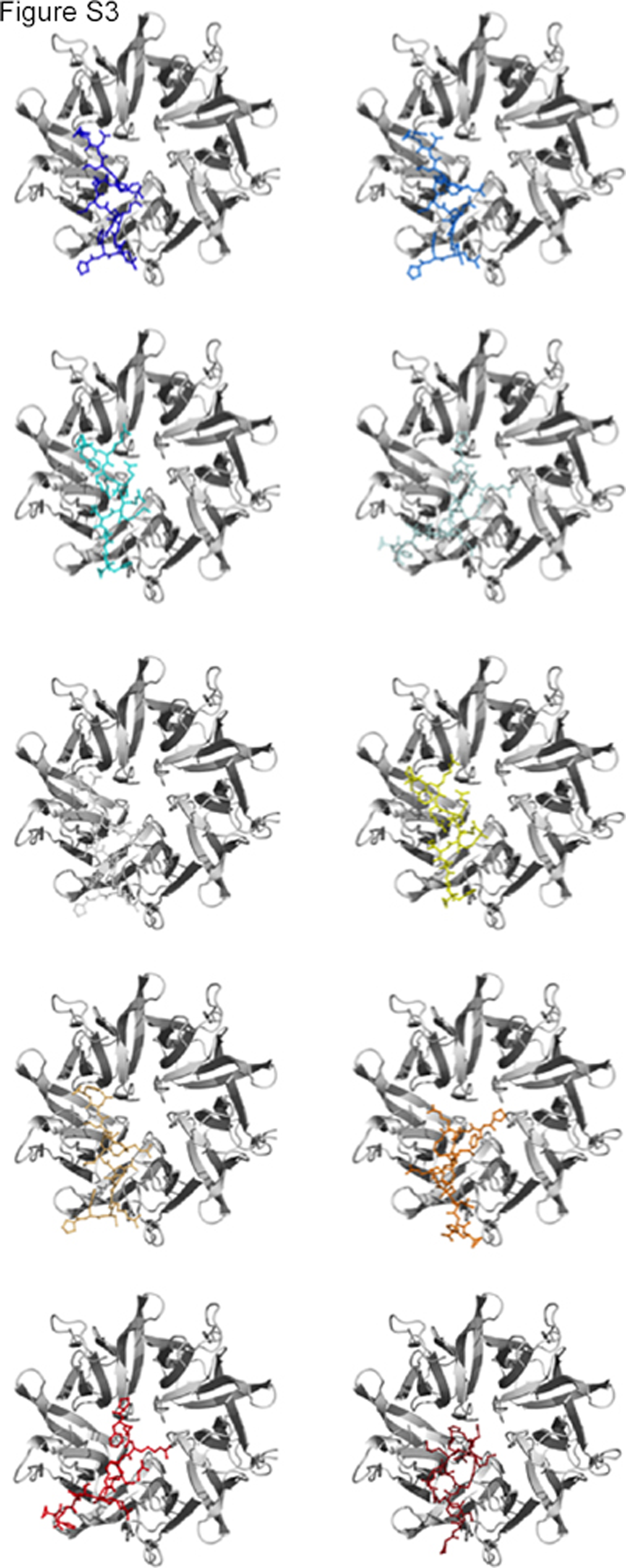

Supplement: Supplementary Figure 3 [file oncsis201778x5.tif]
