# Supplementary material for: Structural investigation of nucleophosmin interaction with the tumor suppressor Fbw7γ
Source: Oncogenesis. 2017 Sep 18;6(9):e379–. doi: 10.1038/oncsis.2017.78 (PMC5623904; doi:10.1038/oncsis.2017.78)
Supplement: Supplementary Figures Legends [file oncsis201778x7.docx]

**Supplementary Figure Legends**

*Figure S1. Interaction analysis of the interaction between CENP-W* and Tat* peptides with Cter-NPM1*

A) Fluorescence maxima as a function of Cter-NPM1 concentrations and B) fluorescence spectra for the CENP-W*-Cter-NPM1 interaction. C) Fluorescence maxima as a function of Cter-NPM1 concentrations and D) fluorescence spectra for the Tat*-Cter-NPM1 interaction.

*Figure S2. Interaction analysis of the interaction between Nter-NPM1 mutants and Fbw7γ* peptide.*

The fluorescence maxima as a function of indicated mutant concentrations are shown for each of the mutants. Data were fitted according to equation 1.

*Figure S3. Docking Results*

The ten top scoring docking poses are shown. They all cluster in the same extended area of the Nter-NPM1 surface with a mean RMSD between poses of 4.3 Å.

*Figure S4. Interaction analysis of Nter-NPM1 mutants with CENP-W* and Tat* peptides*

The fluorescence maxima as a function of indicated mutant concentrations are shown for each of the mutant in the their interaction with A) CENP-W* peptide and B) Tat* peptide, respectively. Data were fitted according to equation 1.
